# Calpain-2 mediates SARS-CoV-2 entry via regulating ACE2 levels

**DOI:** 10.1128/mbio.02287-23

**Published:** 2024-02-13

**Authors:** Qiru Zeng, Avan Antia, Luis Alberto Casorla-Perez, Maritza Puray-Chavez, Sebla B. Kutluay, Matthew A. Ciorba, Siyuan Ding

**Affiliations:** 1 Department of Molecular Microbiology, Washington University School of Medicine, St. Louis, Missouri, USA; 2 Division of Gastroenterology, Department of Medicine, Inflammatory Bowel Diseases Center, Washington University School of Medicine, St. Louis, Missouri, USA; Icahn School of Medicine at Mount Sinai, New York, USA

**Keywords:** SARS-CoV-2, viral entry, antivirals

## Abstract

Since the beginning of the coronavirus disease 2019 (COVID-19) pandemic, much effort has been dedicated to identifying effective antivirals against severe acute respiratory syndrome coronavirus 2 (SARS-CoV-2). A number of calpain inhibitors show excellent antiviral activities against SARS-CoV-2 by targeting the viral main protease (M^pro^), which plays an essential role in processing viral polyproteins. In this study, we found that calpain inhibitors potently inhibited the infection of a chimeric vesicular stomatitis virus (VSV) encoding the SARS-CoV-2 spike protein but not M^pro^. In contrast, calpain inhibitors did not exhibit antiviral activities toward the wild-type VSV with its native glycoprotein. Genetic knockout of calpain-2 by CRISPR/Cas9 conferred resistance of the host cells to the chimeric VSV-SARS-CoV-2 virus and a clinical isolate of wild-type SARS-CoV-2. Mechanistically, calpain-2 facilitates SARS-CoV-2 spike protein-mediated cell attachment by positively regulating the cell surface levels of ACE2. These results highlight an M^pro^-independent pathway targeted by calpain inhibitors for efficient viral inhibition. We also identify calpain-2 as a novel host factor and a potential therapeutic target responsible for SARS-CoV-2 infection at the entry step.

**IMPORTANCE:**

Many efforts in small-molecule screens have been made to counter SARS-CoV-2 infection by targeting the viral main protease, the major element that processes viral proteins after translation. Here, we discovered that calpain inhibitors further block SARS-CoV-2 infection in a main protease-independent manner. We identified the host cysteine protease calpain-2 as an important positive regulator of the cell surface levels of SARS-CoV-2 cellular receptor ACE2 and, thus, a facilitator of viral infection. By either pharmacological inhibition or genetic knockout of calpain-2, the SARS-CoV-2 binding to host cells is blocked and viral infection is decreased. Our findings highlight a novel mechanism of ACE2 regulation, which presents a potential new therapeutic target. Since calpain inhibitors also potently interfere with the viral main protease, our data also provide a mechanistic understanding of the potential use of calpain inhibitors as dual inhibitors (entry and replication) in the clinical setting of COVID-19 diseases. Our findings bring mechanistic insights into the cellular process of SARS-CoV-2 entry and offer a novel explanation to the mechanism of activities of calpain inhibitors.

## INTRODUCTION

High mutation rates of SARS-CoV-2 pose great challenges for antiviral drug development and treatment of COVID-19 patients. Thus far, most antiviral strategies have directly targeted key viral factors involved in the SARS-CoV-2 replication cycle (1). Remdesivir (Gilead) and molnupiravir (Merck) represent two FDA-authorized antiviral drugs that inhibit the SARS-CoV-2 RNA-dependent RNA polymerase (RdRp) (2). In addition to RdRp, the viral main protease (M^pro^) has been a drug target of great interest due to its fundamental role in processing the viral polyproteins. In a series of studies, M^pro^ inhibitors, including Paxlovid (Pfizer), boceprevir, GC376, and various calpain inhibitors, were reported to potently supress SARS-CoV-2 replication in different cell types and in pre-clinical animal models (3, 4).

In a recent study from our group (5), we performed a drug repurposing screen and identified several compounds that potently block SARS-CoV-2 infection. One such compound is MG132, a commonly used 26S proteasome inhibitor. MG132 was previously reported to impair SARS-CoV replication by inhibiting the host cysteine protease m-calpain, also known as calpain-2 (encoded by *CAPN2*), thus functioning through a proteosome-independent pathway (6). Several pieces of evidence led us to hypothesize that host calpain proteases may be required for SARS-CoV-2 infection. First, MG132 inhibited SARS-CoV-2 replication, while ubiquitin-activating enzyme E1 inhibitor PRY-41 and two other proteasome inhibitors bortezomib and lactacystin did not. Second, E-64, which inhibits endosomal cathepsins, papain, and calpain, inhibited SARS-CoV-2 more robustly than chloroquine, which only targets cathepsins and not calpain. Third, calpain inhibitor II and calpeptin suppressed SARS-CoV-2 replication (4, 7) although the mechanism was postulated to be mediated by interfering with activities of M^pro^.

Of note, in our follow-up studies described here, we found that MG132 also exhibited antiviral activities against a chimeric vesicular stomatitis virus (VSV) expressing SARS-CoV-2 spike protein (VSV-SARS-CoV-2) (8) but not expressing M^pro^. The lack of inhibition against wild-type (WT) VSV led us to hypothesize that (1) SARS-CoV-2 spike protein may be an additional viral target of calpain and protease inhibitors and (2) calpain proteins themselves may be crucial host factors for SARS-CoV-2 infection. In this paper, we confirm these hypotheses and identify *CAPN2* as a novel pro-viral host factor that aids in the entry of SARS-CoV-2. We demonstrate that the absence of *CAPN2* reduces viral binding to host cells and RNA production during early steps of the SARS-CoV-2 replication cycle. The findings provide mechanistic insights into the cellular process of SARS-CoV-2 entry and offer an additional explanation to the mechanism of action of calpain inhibitors.

## RESULTS

### MG132 preferentially inhibits the infection of VSV-SARS-CoV-2 but not VSV

**Fig 1:**
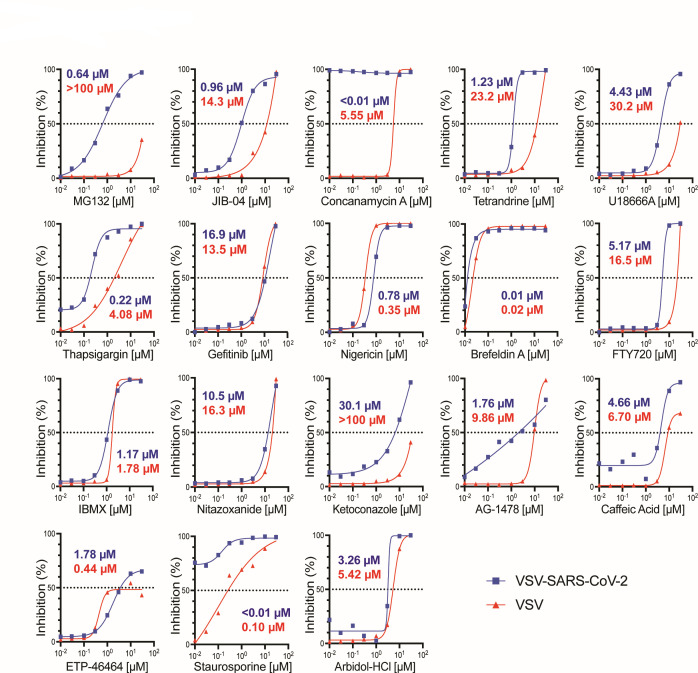
Small-molecule compounds inhibit VSV and VSV-SARS-CoV-2 infection Screening of 18 compounds over a 24-h infection period. MA104 cells were pre-treated with each compound for 1 h at indicated concentrations ranging from 0.01 to 30 µM and then infected for 24 h with either recombinant VSV-SARS-CoV-2 (MOI = 1) or VSV (MOI = 1). Quantified GFP signals are plotted as percentage of inhibition corresponding to dosage. EC_50_ values for each curve are indicated in blue (VSV-SARS-CoV-2) or red (VSV).

In a recent antiviral compound screen that we conducted using a recombinant SARS-CoV-2 mNeonGreen reporter virus (5), multiple compounds efficaciously inhibited viral infection in Vero E6 cells. We validated 18 of the top hits using recombinant VSV eGFP reporter viruses that encode either the SARS-CoV-2 spike protein or the native VSV-G (8). Among the 18 compounds that we tested, most showed a dose-dependent inhibition of VSV-SARS-CoV-2 and VSV infections in MA104 cells (Fig. 1). Nigericin, brefeldin A, and 3-isobutyl-1-methylxanthine (IBMX) had effective concentration to reach 50% inhibition (EC_50_) values lower than 2 µM against both VSV-SARS-CoV-2 and VSV infections (Fig. 1). Nitazoxanide was recently reported to inhibit SARS-CoV-2 infection (9) and we observed similar results (Fig. S1A through C). In contrast, rosmarinic acid, which was speculated to have antiviral activity (10), did not inhibit VSV-SARS-CoV-2 infection (Fig. S1D). In addition, we noticed that MG132, a broad-spectrum proteasome inhibitor, exhibited a 100-fold selectivity in antiviral activities against VSV (EC_50_ of 44.4 µM) and VSV-SARS-CoV-2 (EC_50_ of 0.64 µM) (Fig. 1). Notably, neither of the two reporter viruses express M^pro^ and the only difference lies in the VSV glycoprotein replaced by the SARS-CoV-2 spike protein. These data, together with the previous report of MG132 and SARS-CoV, led us to further test the antiviral activities of MG132 and other calpain inhibitors and their effect on the spike protein.

### Calpain inhibitors strongly inhibit VSV-SARS-CoV-2 infection

**Fig 2:**
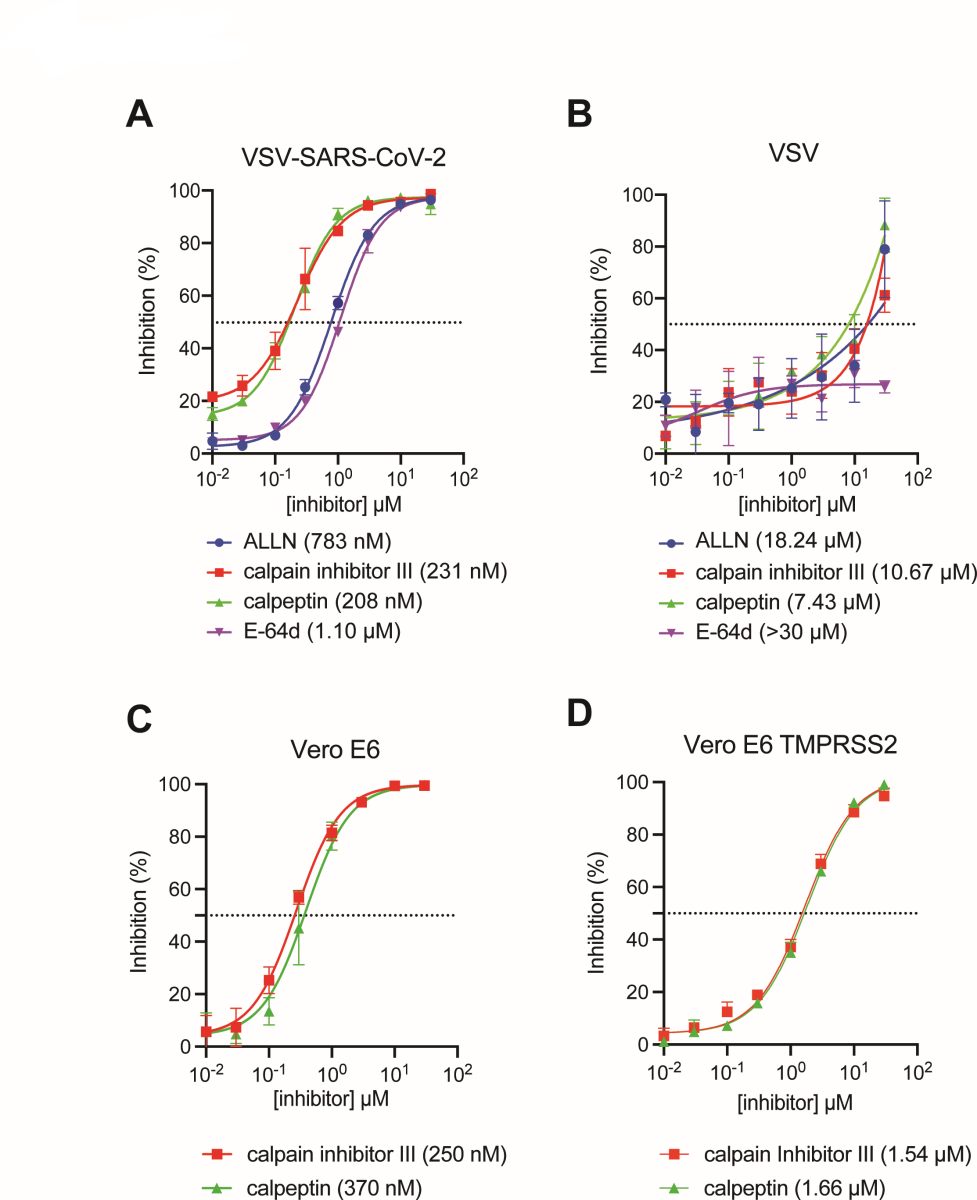
Calpain inhibitors potently inhibit VSV-SARS-CoV-2 infection. (**A**) MA104 cells were pretreated with ALLN, calpain inhibitor III, calpeptin, and E-64d at concentrations ranging from 0.01 to 30 µM for 1 h prior to a 24 h infection by VSV-SARS-CoV-2 (MOI = 1). GFP signals were quantified and plotted as percentage of inhibition corresponding to dosage. EC50s values are as indicated. (**B**) Same as (**B**) except that VSV was used for infection instead of VSV-SARS-CoV-2. (**C**) Same as (**B**) except that Vero E6 cells were used instead of MA104 cells. (**D**) Same as (**B**) except that Vero E6 TMPRSS2 cells were used instead of MA104 cells.

To examine whether MG132 targeting host calpain proteases accounts for the inhibition observed, we tested a set of commercially available calpain inhibitors, including ALLN (also known as MG101 or calpain inhibitor I), calpain inhibitor III, calpeptin, and E-64d since these calpain inhibitors vary in their specificities targeting different members of the calpain family (11). With the exception of calpeptin, none of the inhibitors were cytotoxic even at the highest concentration tested (Fig. S1E). All four calpain inhibitors exhibited potent inhibition against VSV-SARS-CoV-2 with EC_50_ values lower than 1.5 µM (Fig. 2A). Calpain inhibitor III and calpeptin, which are more inhibitory against CAPN2 (11), showed stronger efficacy than ALLN and E-64d, with EC_50_ values of 231 and 208 nM, respectively (Fig. 2A). VSV was reported to not be sensitive to E-64d treatment (12). Consistently, all four calpain inhibitors were substantially less inhibitory against VSV infection (Fig. 2B). For instance, calpain inhibitor III and calpeptin had EC_50_ values of 10.67 and 7.43 µM, respectively, indicating a more than 30-fold increase when compared to VSV-SARS-CoV-2 (Fig. 2B). In addition to MA104 cells, the antiviral activities of calpain inhibitor III and calpeptin against VSV-SARS-CoV-2 were confirmed in Vero E6 cells, with EC_50_ values lower than 500 nM (Fig. 2C). Interestingly, the inhibitory effects of the compounds were reduced by at least five fold in Vero E6 cells expressing human TMPRSS2 (Fig. 2D), suggesting that the mechanism of action could be associated with the virus entry pathway. Notably, VSV-SARS-CoV-2 does not encode M^pro^, the key therapeutic target identified in many previous protease inhibitor studies. Therefore, the contrasting results of inhibition of VSV-SARS-CoV-2 compared to VSV and the more pronounced inhibitory effects seen with calpain inhibitor III and calpeptin prompted us to hypothesize that these calpain inhibitors may play a role in interfering with the activities of the SARS-CoV-2 spike protein by inhibiting the host gene *CAPN2*.

### VSV-SARS-CoV-2 infection is significantly reduced in *CAPN2* knockout cells

**Fig 3:**
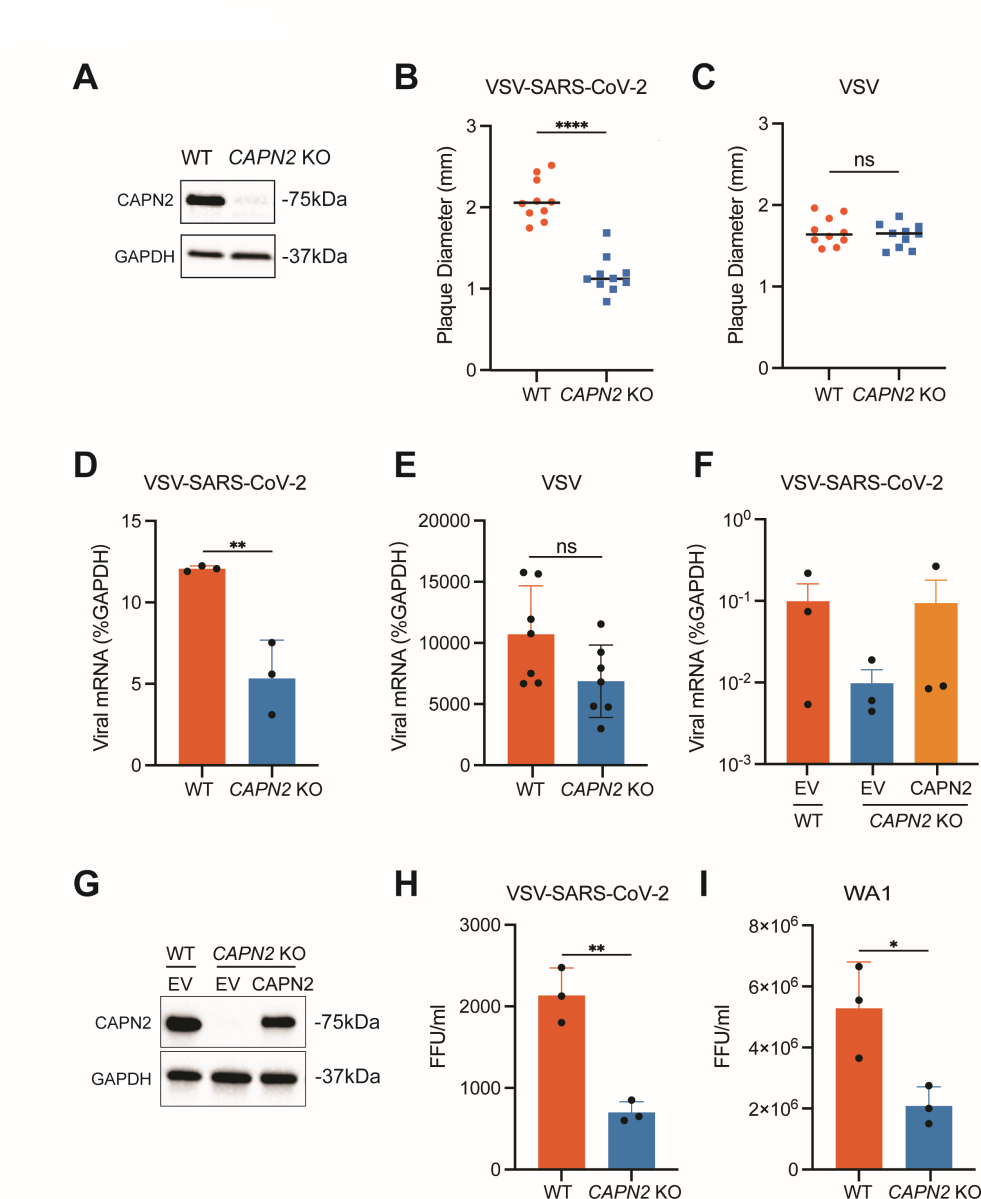
*CAPN2* KO cells have reduced VSV-SARS-CoV-2 infection. (**A**) Cell lysates of WT and *CAPN2* KO MA104 cells were harvested, and the protein levels of CAPN2 and GAPDH were measured by western blot. (**B**) Plaque assays of VSV-SARS-CoV-2 were performed in MA104 cells. Images were taken at 72 hpi when clear-shaped plaques were observed. Ten plaques from each sample were selected and measured using microscopy. (**C**) Same as (**B**) except VSV was used instead and images were taken at 48 hpi. (**D**) Viral mRNA production in WT and *CAPN2* KO cells upon infection of VSV-SARS-CoV-2 at an MOI of 0.01 at 24 hpi. Viral levels were calculated via RT-qPCR and shown as percentage of GAPDH. Each bar represents three replicates. (**E**) Viral mRNA production in WT and *CAPN2* KO cells upon infection of VSV at an MOI of 0.01 at 24 hpi. Viral levels were calculated via RT-qPCR and shown as percentage of GAPDH. Each bar represents seven replicates. (**F**) Viral mRNA production in WT, *CAPN2* KO, and put-back cells upon infection of VSV-SARS-CoV-2 at an MOI of 0.01 at 24 hpi. Three replicates were represented. (**G**) WT and *CAPN2* KO cells were transfected with empty vector (ev) controls. *CAPN2* KO cells were transduced with lentiviral vectors and further transfected with a CAPN2-encoding plasmid. CAPN2 protein levels were confirmed by western blot. (**H**) FFU assay showing titers of produced VSV-SARS-CoV-2 at 24 hpi after inoculation at an MOI of 0.1. Supernatant and infected cells were harvested and titrated on MA104 cells. Each bar represents three replicates. (**I**) FFU assay showing titers of produced WA1 at 48 hpi after inoculation at an MOI of 0.1. Supernatant and infected cells were harvested and titrated on Vero E6 cells. Each bar represents three replicates.

To directly investigate the role of the host gene *CAPN2* in SARS-CoV-2 infection, we genetically knocked out *CAPN2* by lentivirus-mediated CRISPR/Cas9 in MA104 cells, which express the endogenous ACE2 receptor that is necessary for virus entry. The *CAPN2* knockout (KO) efficiency was validated by western blot (Fig. 3A). We performed standard plaque assays of VSV-SARS-CoV-2 infections at MOIs of 1, 0.1, 0.01, and 0.001 in WT and *CAPN2* KO cells. Ten plaques from each group were selected, and the diameters of plaque sizes were quantified. The plaques of VSV-SARS-CoV-2 formed in the KO cells had an average of diameter of 1 mm, significantly smaller than the 2 mm observed in the WT cells (Fig. 3B). To test whether this phenotype was associated with the spike protein, we performed similar plaque assays using VSV. No significant difference in sizes of VSV plaques was observed between WT and KO cells (Fig. 3C), suggesting that CAPN2 promotes the replication of VSV-SARS-CoV-2 but not VSV by acting on the spike protein or facilitating spike protein related functions. Based on the findings above, we further generated single clonal *CAPN2* KO MA104 cells, which were confirmed by Sanger sequencing (Fig. S2A). The growth rate of *CAPN2* KO cells was similar to that of the WT cells (Fig. S2B and C). Next, we examined the intracellular viral mRNA levels at 24 hpi followed infection by VSV-SARS-CoV-2 or VSV. VSV-SARS-CoV-2 but not VSV mRNA was significantly decreased in *CAPN2* KO cells, suggested a pro-viral role of CAPN2 in VSV-SARS-CoV-2 infection (Fig. 3D and E). To validate that the observed phenotype was specifically due to the loss of CAPN2, we re-introduced the expression of a CRISPR/Cas9 cleavage-resistant version of CAPN2 into the *CAPN2* KO cells. The *CAPN2* add-back cells had similar CAPN2 protein levels and restored VSV-SARS-CoV-2 infectivity compared to the WT cells (Fig. 3F and G). We then sought to quantify the infectious virus titers of VSV-SARS-CoV-2 and a clinical isolate of infectious SARS-CoV-2 (2019-nCoV/USA-WA1/2020 strain). In addition to the plaque diameter, production of infectious VSV-SARS-CoV-2 and SARS-CoV-2 was significantly attenuated in *CAPN2* KO cells, revealed by focus-forming assays (Fig. 3H and I). Based on the results above, *CAPN2* seems to facilitate the infections of VSV-SARS-CoV-2 and SARS-CoV-2 WA1.

### CAPN2 is required for an early step of the SARS-CoV-2 replication cycle

**Fig 4:**
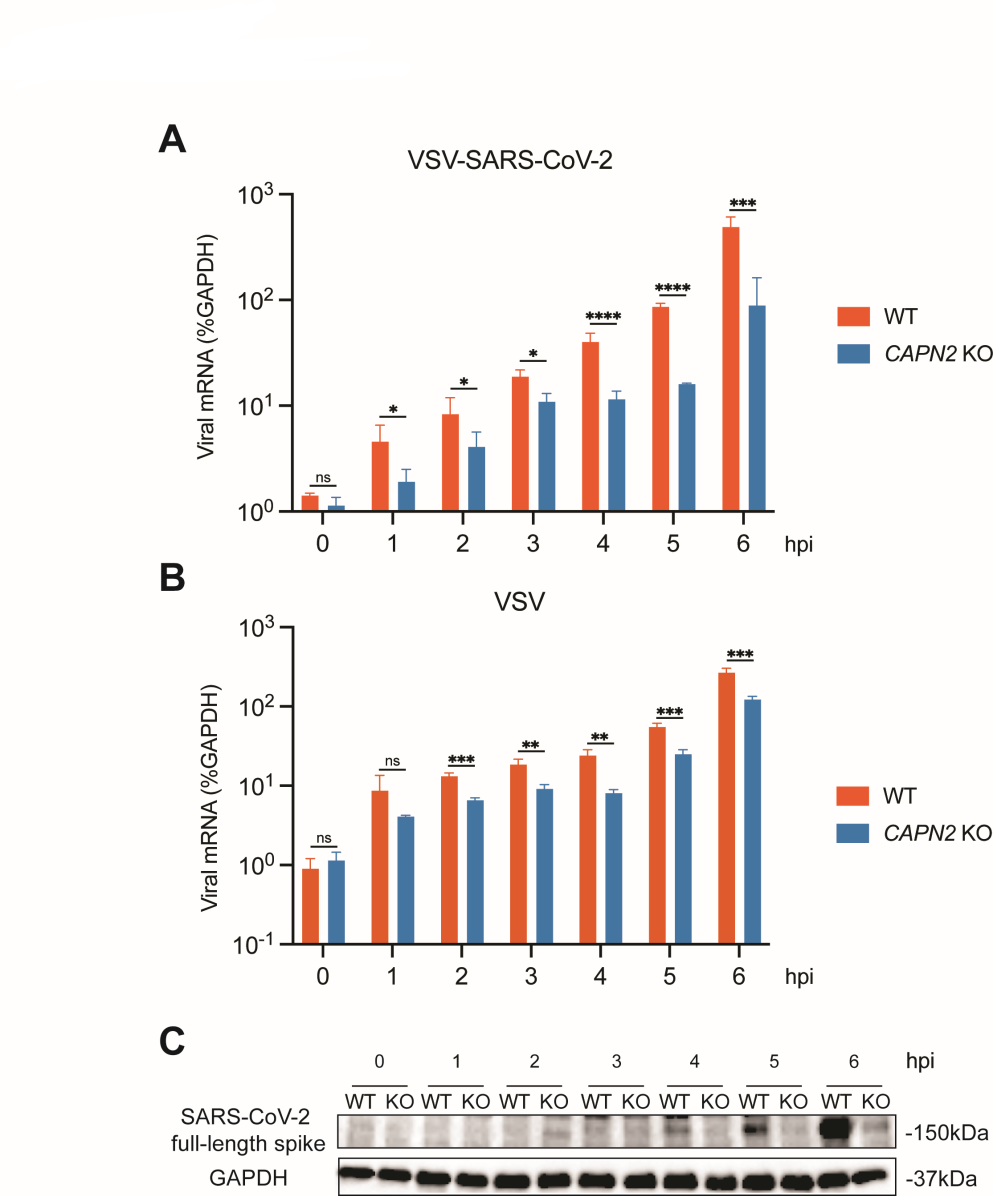
*CAPN2* deletion reduces SARS-CoV-2 infection. (**A**) Viral mRNA production at early time points post infection by VSV-SARS-CoV-2 (MOI = 1) in WT and *CAPN2* KO MA104 cells. Cells were harvested at 0, 1, 2, 3, 4, 5, 6 h post infection for RNA extraction followed by RT-qPCR analysis. Viral mRNA levels are shown relative to those of GAPDH. Each bar represents three replicates. (**B**) Same as (**A**) except cells were infected by VSV instead. (**C**) Same as (**A**) except that SARS-CoV-2 full-length spike levels were measured by western blot instead.

In order to determine the specific step of the replication cycle where CAPN2 functions to aid viral infection, we infected both WT and *CAPN2* KO cells with VSV-SARS-CoV-2 and examined viral protein expression within a single replication cycle at 8 hpi using flow cytometry (Fig. S3). GFP was used as a proxy for the levels of infection. Compared to the uninfected mock, 45.3% of the WT cells were infected by VSV-SARS-CoV-2, whereas the infectivity dropped to 26.4% in the *CAPN2* KO cells. Of note, such difference was not observed with VSV infection (Fig. S3). We next sought to pinpoint the time point when CAPN2 exerts its pro-viral effect. A time-course experiment was performed by infecting WT and KO cells with VSV-SARS-CoV-2 and examining intracellular viral RNA levels at 1–6 h post infection by RT-qPCR (Fig. 4A). Our results show that as early as 1 h post infection, significantly lower viral mRNA levels were observed in the KO cells than those in the WT cells (Fig. 4A). Similar reduction was seen throughout the course of infection (Fig. 4A). While VSV mRNA levels were also negatively impacted in the absence of CAPN2, the difference was not as prominent as in the case of VSV-SARS-CoV-2 infection (Fig. 4B). SARS-CoV-2 spike protein levels as the nascent protein synthesis was first visible starting at 4 h post infection in WT cells, whereas it was barely detectable at 6 h post infection in *CAPN2* KO cells (Fig. 4C).

### CAPN2 regulates SARS-CoV-2 infection in a spike-dependent and viral strain-specific manner

**Fig 5:**
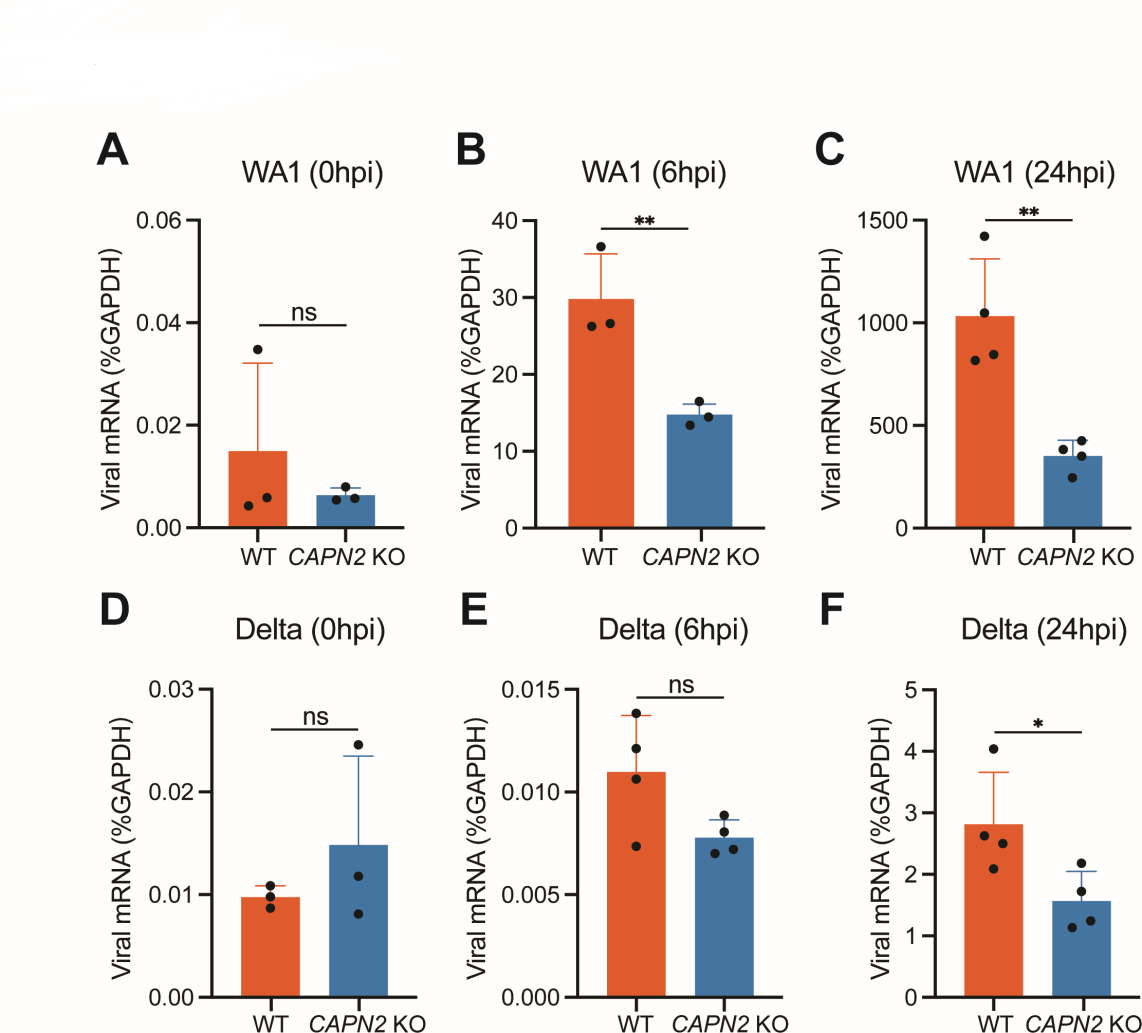
CAPN2 deletion yielded varied impact on different strains of SARS-CoV-2 infection. (**A**) Viral mRNA production in WT and *CAPN2* KO cells upon infection of WA1 at an MOI of 0.1 and harvested at 0 hpi for RNA extraction as the input. Viral mRNA levels were calculated via RT-qPCR and shown as percentage of GAPDH. (**B, C**) Same as A except that RNA was harvested at 6 hpi and 24 hpi, respectively. (**D–F**) Same as (**A–C**) except that cells were infected with SARS-CoV-2 Delta variant instead. For (**A, B, D**), each bar represents three replicates. For (**C, E, F**), each bar represents four replicates.

Concurrent SARS-CoV-2 variants accumulate multiple mutations in the spike protein that result in enhanced transmission and antibody evasion (13). To that end, we tested SARS-CoV-2 ancestral WA1 strain and Delta and Omicron variants. All viruses were propagated and carefully confirmed by deep sequencing (Table S1). We performed infection and harvested intracellular viral mRNA for quantification at different time points. Consistent with the FFU results (Fig. 3I), we found a statistically significant decrease in the SARS-CoV-2 WA1 mRNA production in *CAPN2* KO cells at 6 and 24 hpi (Fig. 5A through C). In comparison, the loss of CAPN2 had a weaker impact on the replication of Delta variant (Fig. 5D through F). In addition, we examined the impact of CAPN2 on viral mRNA production of Omicron and a recombinant SARS-CoV-2 strain with spike mutations in three key residues E484K, N501Y, and D614G (14) with the same assay (Fig. S4). The Omicron variant was less dependent on CAPN2 than WA1 (Fig. S4A through C). Although the triple variant trended lower in *CAPN2* KO cells, similar to the Delta variant, it was not significant until 24 hpi beyond one replication cycle (Fig. S4D and E). Taken together, these data suggest that the effect of CAPN2 on SARS-CoV-2 is potentially dependent on the nature of spike proteins and how they engage with the cellular receptors.

### CAPN2 promotes SARS-CoV-2 binding to host cells

**Fig 6:**
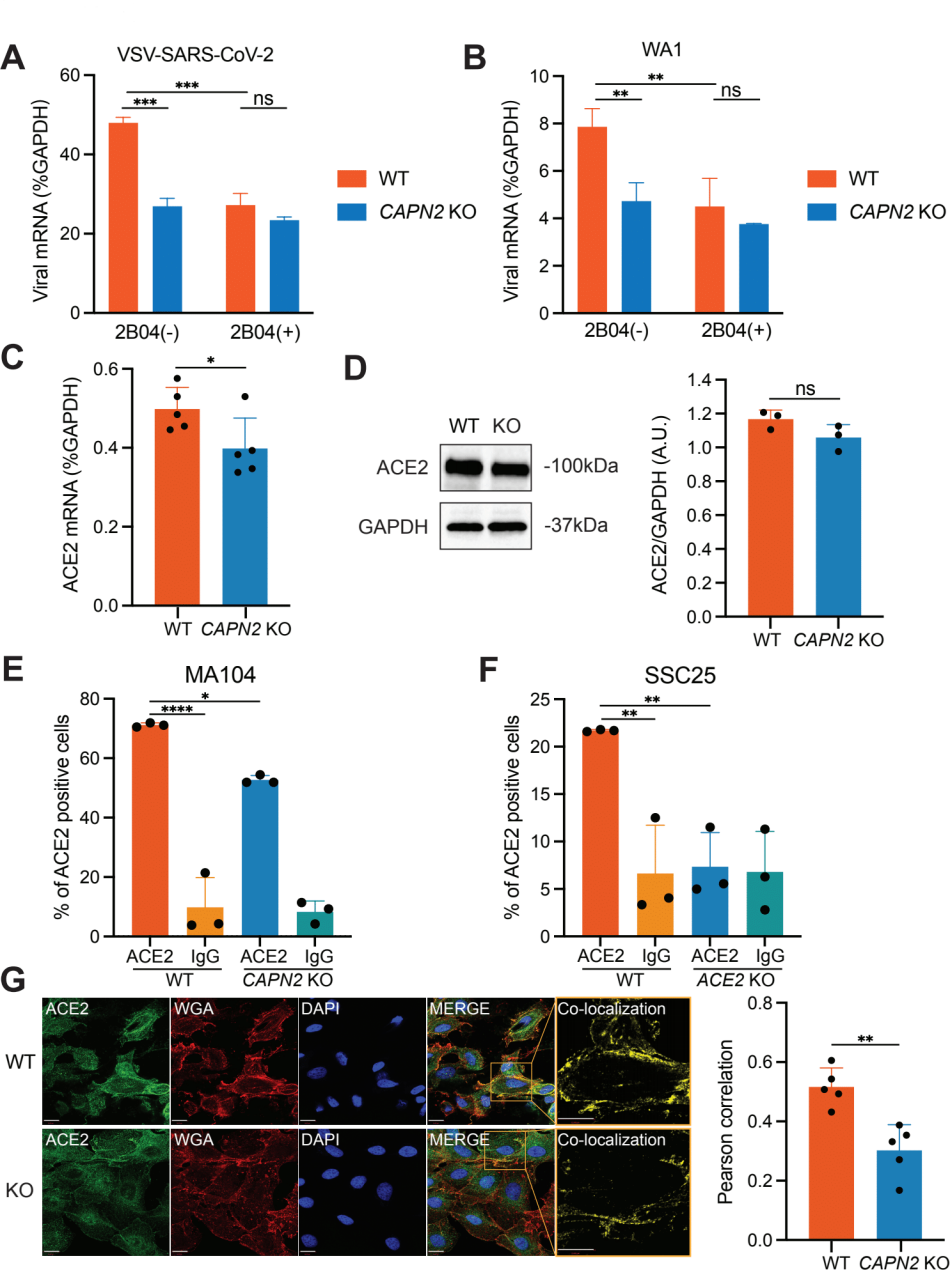
CAPN2 enhances ACE2 surface levels and spike-mediated virus attachment. (**A**) Cold binding assay with VSV-SARS-CoV-2. WT and *CAPN2* KO MA104 cells were infected by VSV-SARS-CoV-2 alone (MOI = 20) or virus pre-incubated with a spike-targeted neutralizing antibody 2B04 (10 µg/mL) for 1 h on ice followed by RNA extraction and RT-qPCR. Viral mRNA levels were measured and normalized to those of GAPDH. (**B**) Same as (**A**) except that SARS-CoV-2 strain WA1 was used instead. (**C**) ACE2 mRNA levels of WT and *CAPN2* KO cells. WT and *CAPN2* KO MA104 cells were harvested for RNA extraction followed by RT-qPCR analysis. The mRNA levels of ACE2 were measured and normalized to those of GAPDH. (**D**) Bulk ACE2 protein levels in WT and *CAPN2* KO cells. WT and *CAPN2* KO MA104 cells were harvested for western blot examining the levels of ACE2 and GAPDH. ACE2 protein levels were quantified from three repeated experiments described above, shown as ratio to GAPDH in arbitrary units (A.U.). Intensities of bands were quantified using ImageJ. (**E**) Surface ACE2 levels of WT and *CAPN2* KO MA104 cells shown in percentages of ACE2 positive cells, measured and quantified by flow cytometry analysis. Cells were detached by accutase and stained with an anti-ACE2 antibody or an IgG rabbit antibody control. Each bar represents three biological replicates. (**F**) Surface ACE2 levels of WT and *ACE2* KO SSC-25 cells shown in percentages of ACE2 positive cells, measured and quantified by flow cytometry analysis. Samples were prepared as they were in (**E**). Each bar represents three biological replicates. (**G**) Confocal analysis of surface levels of ACE2 in WT and *CAPN2* KO cells. WT and *CAPN2* KO MA104 cells were fixed and stained for surface glycoprotein (red, WGA), ACE2 (green), and nucleus (blue, DAPI). Co-localization of ACE2 and WGA-stained cell membrane glycoprotein in WT and *CAPN2* KO MA104 cells was quantified by Pearson correlation measurements. Scale bars: 16 µm (ACE2, WGA, DAPI, merge), 13 µm (co-localization). Each bar represents five replicates.

As suggested by the reduced levels of viral mRNA levels as early as 1 hpi (Fig. 4A), we reasoned that CAPN2 may play an important role at a very early time point of the SARS-CoV-2 replication cycle, i.e., virus binding and entry, which spike protein mediates. To test this hypothesis, and took advantage of a classical cold binding assay (15) using VSV-SARS-CoV-2 to assess whether viral adsorption is negatively impacted by the lack of CAPN2. The assay was performed at 4°C to allow virus binding to host cells but limit the energy required for virus endocytosis to gain entry into cells, followed by extensive wash and RT-qPCR analysis. As a positive control, we included a neutralizing antibody (2B04) that targets spike from the ancestral SARS-CoV-2 WA1 strain (16), the preincubation of which significantly reduced binding of the virus (Fig. 6A). Importantly, we found that the viral RNA levels from the virions bound to the KO cells were comparable to those in WT cells in the presence of antibody incubation and significantly lower than those in the WT cells without the antibody (Fig. 6A). Similar binding defects in the KO cells were observed with WT SARS-CoV-2 WA1 strain (Fig. 6B). Viral binding in the KO cells were essentially reduced to the background levels similar to the antibody incubation controls (Fig. 6B).

To dissect the mechanism underlying reduced viral binding in the absence of CAPN2, we tested potential spike cleavage by CAPN2 given its role as a protease. We co-transfected HEK293 cells stably expressing human ACE2 with spike derived from WA1 strain, along with EGFP (control), CAPN2, transmembrane serine protease 2 (TMPRSS2), or furin, the major proteases known to cleave spike for efficient entry (17). The cell lysates were harvested, and the intensities of the full-length spike and its cleaved product S2 fragment were quantified (Fig. S5A). The spike cleavage efficiency was plotted as percentage of the cleavage product within overall spike protein levels. In this assay, overexpression of V5-tagged CAPN2 did not lead to significant spike cleavage more than EGFP, the negative control, when compared to other host proteases such as TMPRSS2 and furin (Fig. S5B).

Next, we examined the levels of SARS-CoV-2 cellular receptor ACE2 in WT and *CAPN2* KO cells. Interestingly, although the transcriptional level of ACE2 was slightly but statistically lower in *CAPN2* KO cells (Fig. 6C), this difference was not reflected on the bulk protein level (Fig. 6D). Of note, surface ACE2 levels, measured by flow cytometry, were significantly decreased in *CAPN2* KO MA104 cells, compared to WT cells (Fig. 6E). To verify the validity of our assay, we generated WT and *ACE2* KO SCC-25 cells, a human tongue epithelial cell line (Fig. S6A). There were almost non-detectable levels of surface ACE2 in *ACE2* KO SSC-25 cells (Fig. 6F). Surface ACE2 level was also quantified by mean florescence intensity, and a similar trend was observed comparing WT and KO cells (Fig. S6B and C). In addition, we observed a reduced amount of surface ACE2 present in *CAPN2* KO cells when the cells were scraped off instead of accutase treatment during the detachment (**Fig. S6D**). We further confirmed this finding by staining for ACE2 subcellular localization in WT and KO cells. Consistently, we observed much higher levels of surface ACE2 in WT cells co-localizing with wheat germ agglutinin (WGA) at the plasma membrane by confocal microscopy in contrast to higher levels of intracellular ACE2 seen in the KO cells (Fig. 6G). Co-localization analysis of ACE2 and WGA, indicated by yellow signals in the inset images, showed that the surface ACE2 levels were significantly reduced in the *CAPN2* KO cells (Fig. 6G). Collectively, these data suggest that CAPN2 positively regulates the presence of ACE2 at the cell surface, thus enhancing spike-mediated SARS-CoV-2 binding and viral infectivity.

### CAPN2 levels positively correlate with viral infection in primary human intestinal epithelial cells

To investigate if CAPN2 plays a pro-viral role in physiologically relevant human cell types, we turned to the human small intestinal enteroids, which can be productively infected by SARS-CoV-2 (18, 19). Primary human ileal enteroids were derived from six donors, and we determined the CAPN2 transcript levels that are affected by individual genetic heterogeneity. We found that two of these human enteroids, 217 and 242, had higher levels of CAPN2, ~7- and ~3-fold, respectively, than the other four samples (Fig. S7A). We further tested the hypothesis that higher expression levels of CAPN2 leads to increased susceptibility to coronavirus infection. To that end, we infected human enteroids with VSV-SARS-CoV-2 and performed a correlation analysis of CAPN2 mRNA levels and infectivity. We found a positive correlation between the levels of VSV-N and the expression of CAPN2 (Fig. S7B). Likewise, there was a positive correlation between the GFP reporter expression and CAPN2 levels (Fig. S7C). Thus, our findings show that a high expression of CAPN2 in primary human cells is associated with increased susceptibility to infection.

## DISCUSSION

SARS-CoV-2, like SARS-CoV, employs the spike protein that engages surface ACE2 to bind to host cells and is primed by TMPRSS2 and TMPRSS4 (18), as well as host cysteine proteases cathepsins B and L for entry into host cells (20). Many proteases contribute to viral entry of SARS-CoV-2 and the development of immunopathology during COVID-19 diseases (18, 20–23). In this study, we uncovered the host protease CAPN2 as a novel host factor that aides the infection of SARS-CoV-2.

CAPN2 plays a major role in cancer-related cell proliferation (24, 25). Although participation of calpains in virus infections has not been well understood, several published studies indicate pro-viral functions of the host gene CAPN2. CAPN2 expression was discovered to be an indicator of level of hepatic fibrosis during hepatitis B virus infection (26). Additionally, CAPN2 enhances replication of echovirus 1 at a late stage step during the viral replication cycle (27), and CAPN2 promotes coxsackievirus entry into host cells (28). In our study, we utilized the recombinant VSV-SARS-CoV-2 as a surrogate for SARS-CoV-2 and observed viral inhibition by calpain inhibitors through a series of experiments using chemical inhibitors, genetic knockouts, and classical virological approaches. Unlike the literature describing the direct role of CAPN2 in viral entry and assembly, we show that CAPN2 also promotes SARS-CoV-2 infection by acting early to aid virus binding (Fig. 6). Our new data add to the vast literature of calpain inhibitors harboring antiviral activities by targeting M^pro^. It is likely that these calpain inhibitors have dual mechanisms of action and additionally blocks viral entry in an M^pro^-independent manner. By measuring surface ACE2 levels in both WT and *CAPN2* KO MA104 cells by flow cytometry, we discovered the lack of CAPN2 is accompanied with a moderate reduction of cell surface ACE2 (Fig. 6E). Further studies are needed to understand whether CAPN2 modulates ACE2 endosomal trafficking, recycling, or degradation. It is worth pointing out that CAPN2 seems to affect VSV infection to some extent (Fig. 3E and 4B), which utilizes a different cellular receptor LDLR. We also noticed that when using cell scraping as an alternative method to detach cells for flow cytometry analysis, the peak of IgG control is slightly shifted in the *CAPN2* KO cells (Fig. S6D), suggesting that the Fc receptor level may be altered as well in these cells. It would be of interest to determine in future work how surface proteins other than ACE2 are modulated by CAPN2.

Our current study has a number of limitations. The calpain inhibitors are not tested in primary human airway epithelial cells and their therapeutic utility is not yet explored to inhibit SARS-CoV-2 infection in relevant animal models. Another caveat is the frequency of mutations in the spike protein in SARS-CoV-2 strains. The pro-viral role of CAPN2 in SARS-CoV-2 infection seems to be viral strain-specific (Fig. 5; Fig. S4). It is conceivable, however, that this is largely due to the nature of the interaction of spike proteins with host cell surface receptors during early time points in the replication cycle. For instance, the more recent Omicron variant, whose spike protein has a higher affinity to ACE than WA1 spike (29), is less dependent on the ACE2 density on cell surface and less likely to be affected by CAPN2 that regulates ACE2 surface levels. Nonetheless, our findings highlight a novel function of CAPN2 in mediating SARS-CoV-2 entry and offer an alternative explanation to the protective efficacy of calpain inhibitors independent of blocking M^pro^ activities. To our knowledge, although there are studies of host factors that regulate ACE2 mRNA and protein levels (30, 31), this is the first report of an ACE2 surface level modulator that dictates the outcome of SARS-CoV-2 infection.

## MATERIALS AND METHODS

### Reagents, cells, and viruses

#### Reagents

MG132 (Selleckchem, S2619), gefitinib (Selleckchem, S1025), nigericin (InvivoGen, tlrl-nig/NIG-36-01), brefeldin A (Cell Signaling Technology, 9972S), FTY720 (Santa Cruz Biotechnology, sc-202161A), IBMX, concanamycin A (Enzo Life Sciences, ALX-380-034-C025), tetrandrine (Selleckchem, S2403), U18666A (Cayman Chemical, 10009085), ETP-46464 (Selleckchem, S8050), JIB-04 (Tocris, 4972), nitazoxanide (COVID Box, MMV688991), ketoconazole (COVID Box, MMV637533), AG-1478 (Selleckchem, S2728), caffeic acid (Selleckchem, S7414), thapsigargin (Cell Signaling Technology, 1278S), staurosporine (Cell Signaling Technology, 9953S), and arbidol-HCl (Selleckchem, S2120). Calpain Inhibitor set includes ALLN, calpain inhibitor III, calpeptin, and E-64d used in the viral inhibition assays (208733-1SET, Sigma-Aldrich).

#### Cells

MA104 cells (ATCC, CRL-2378.1) were cultured in M199 medium (Thermo Scientific, 11150067) supplemented with 10% fetal bovine serum (Avantor VWR, 89510-186) and 1× Penicillin-Streptomycin-Glutamine (Thermo Scientific, 10378016). *CAPN2* KO MA104 cells were cultured in complete M199 medium with the addition of puromycin (10 µg/mL) for selection (single-guide RNA sequence: TGATCCGCATCCGAAATCCC). Primers for genomic DNA PCR and Sanger sequencing are as followed: forward: 5′-AAGTTCAGAGGTGAAGCG-3′, reverse: 5′-GGAAGGTGGGGTACATTT-3′. CAPN2 plasmid was purchased from DNASU (clone ID: HsCD00446380), site-directed mutagenesis primers to construct CRISPR/Cas9 cleavage-resistant CAPN2 are as followed: forward: 5′-CTTCTCCCCAGGGGTTTCGGATACGTATCAGTTTCTGT-3′, reverse: 5′-ACAGAAACTGATACGTATCCGAAACCCCTGGGGAGAAG-3′. Vero E6 cells were cultured in DMEM (Thermo Scientific, 11965118) supplemented with 10% fetal bovine serum (FBS) and 1× Penicillin-Streptomycin-Glutamine. Vero E6 TMPRSS2 cells were cultured in DMEM with the same supplements as Vero E6 cells, with 10% blasticidin. SSC-25 cells were cultured in DMEM/Hams F-12 50/50 (Corning, 10-092-CV) with 10% FBS. SSC-25 ACE2 KO SSC-25 cells were cultured in complete DMEM-F12, selected in 2 µg/mL puromycin (single-guide RNA sequence: GTACTGTAGATGGTGCTCAT), and maintained with 1 µg/mL puromycin.

#### Viruses

Recombinant VSV-eGFP (32) and VSV-eGFP-SARS-CoV-2 were previously described (8). WT SARS-CoV-2 clone of the 2019n-CoV/USA_WA1/2020 (WA1/2020) strain, Delta variant (hCoV-19/USA/PHC658/2021), Omicron BA.1 variant (hCoV-19/USA/MD-HP20874/2021), and SARS-CoV-2 containing three point mutations in the spike gene E484K, N501Y, D614G were obtained from Pei-Yong Shi lab (33, 34). Virus stocks were propagated in Vero-E6 TMPRSS2 cells obtained from Sean Whelan lab (Table S1). Viral stock was harvested in Trizol and RNA was extracted according to manufacturer’s protocol. SARS-CoV-2 sequences were enriched using the ARTIC v4.1 primer set for SARS-CoV-2 viral enrichment and sequenced on the Illumina NovaSeq platform. Output sequences were trimmed and aligned with the SARS-CoV-2 reference (NC_045512.2) by The Genome Technology Access Center at Washington University in St. Louis.

### Inhibitor screen

Cells were seeded in 96 well plates. When they reached 80%–90% confluency, they were pretreated with indicated compounds at desired concentrations for 1 h, followed by virus infection for 24 h with the compound present. The cells were then washed and placed in clear PBS for Typhoon imager scanning. Co-encoded GFP serves as an indicator of infection level as the imager detects fluorescent signals. Darker color corresponds to more intense signals and, therefore, higher level of infection. The typhoon images were then processed using ImageJ for quantification of infection level.

### Cell cytotoxicity assay

The cytotoxicity level of calpain inhibitors were determined using the Cell Counting Kit 8 (Abcam, ab228524). Cells in 96-well plates were treated with inhibitors of interest at concentrations within a range from 0.1 to 300 µM at 37°C for 25 h. Fresh medium containing 10 µL of WST-8 substrate was added to each well to replace the inhibitor-containing medium. After 2 h incubation at 37°C protected from the light, absorbance at 460 nm was measured by BioTek ELx800 Microplate Reader and processed by Gen5 software.

### Plaque assay

MA104 cells were plated, grew to confluency in 6-well plates, and were infected with serial diluted viruses in serum-free M199 medium at 37°C for 1 h. Afterward, virus inoculum was replaced with warm agarose mixed with 2× M199 at 1:1 ratio. At 72 h post infection, GFP signals in the plates were scanned by Amershad Typhoon 5 (GE) and plaque sizes were quantified by the ECHO microscope (32).

### RNA extraction and quantitative PCR

RNA extraction were performed using QIAGEN RNeasy Mini kit (QIAGEN, 74104) per manufacturer’s instructions. For WT SARS-CoV-2 and triple variant E484K, N501Y, D614G infections, viral RNA was extracted using TRIzol (Invitrogen, 15596018) and chloroform following the product protocol. Viral mRNA levels (VSV-N forward primer: 5′- GATAGTACCGGAGGATTGACGACTA-3′, VSV-N reverse primer: 5′-TCAAACCATCCGAGCCATTC-3′, SARS-CoV-2 N primer 1: 5′-ATGCTGCAATCGTGCTACAA-3′, primer 2: 5′-GACTGCCGCCTCTGCTC-3′, probe: 5′-/FAM/TCAAGGAACAACATTGCCAA/TAMRA/-3′) were examined by real-time RT-PCR using High Capacity cDNA Reverse Transcription kit (Applied Biosystems, 4368813) and AriaMX (Agilent) with 12.5 µL of either SYBR Green master mix (Applied Biosystems, 4367659) or Taqman master mix (Applied Biosystems, 4444557), reaching a total reaction volume of 25 µL. Expression of each gene was normalized to the expression of housekeeping gene GAPDH as previously described (35). CAPN2 qPCR forward primer: 5′-AAGTAGCGGAAGCCTACAGAAAC-3′, reverse primer: 5′-ATCTTCATGCCGTCTGGTCAG-3′.

### Western blot

Cells were washed with PBS and lysed by RIPA buffer (Thermo Scientific, 89901) supplemented with 100× protease inhibitor cocktail and phosphatase inhibitor (Thermo Scientific, 78420), followed by a 10-min incubation on ice. Cell lysates were then subjected to centrifugation at 13,500 RPM for 10 min at 4°C to remove cell debris and chromatids. The protein samples were then boiled in 2× Laemmli Sample Buffer (Bio-Rad, #1610737EDU) containing 5% β-mercaptoethanol at 95°C for 5 min. Prepared samples were run in 4%–12% gels and transferred onto nitrocellulose membranes. Membranes were blocked in 5% BSA in TBS +0.1% Tween-20 (TBST) at room temperature before incubation at 4°C overnight with primary antibodies: SARS-CoV-2 spike RBD (Sino Biological, 40592-T62), GAPDH (BioLegend, 631402), calpain-2 (Cell Signaling Technology, 2539), ACE2 (R&D Systems, MAB933), S2 (Sino Biological, 40590-T62), and V5 (Cell Signaling Technology, 13202S). Membranes were then washed three times with TBST and incubated in secondary antibodies accordingly: anti-mouse HRP-linked IgG (Cell Signaling Technology, 7076S) or anti-rabbit HRP-linked IgG (Invitrogen, A27036) diluted in 5% BSA in TBST at room temperature for 1 h. After the secondary antibody incubation, the membranes were washed three times with TBST and visualized by using Chemi-Doc imaging system (Bio-Rad).

### Focus forming unit assays

MA104 cells and Vero E6 cells were seeded in 96-well plates and used when reached close to 100% confluency. Harvested supernatants from infected cells were serial diluted and inoculated onto prepared 96-well plates with MA104 cells or Vero E6 cells. After 1 h incubation at 37°C, the inoculum was removed and replaced with fresh media and the plate was incubated at 37°C overnight for 16–18 h. Cells infected with VSV-SARS-CoV-2 was directly taken under the microscope and examined for GFP. Cells infected with replication-competent SARS-CoV-2 WA1 was fixed with 4% PFA for 15 min, washed twice with PBS, and permeabilized with 0.1% Triton-X100 in PBS for 10 min. After two washes with PBS with 0.1% Tween-20 (PBST), the cells were blocked for 1 h at room temperature using 1%BSA with 10%FBS in PBST. Cells were incubated at 4°C overnight with SARS-CoV-2 nucleocapsid antibody (Sino Biological, 40588-T62) in PBST +1% BSA, diluted at 1:1,000. Post primary antibody incubation, cells were washed twice in PBST and incubated for 45 min in dark with anti-rabbit IgG HRP-linked antibody (Cell Signalling, 7074S) in PBST +1% BSA, diluted at 1:1,000. Finally, the cells were incubated with AEC substrate (Vector Laboratories, SK-4200) for antigen labeling.

#### Flow cytometry

After infection, VSV-SARS-CoV-2 and VSV infected cells were detached and collected by centrifugation, washed once with PBS, and fixed with BD Cytofix/Cytoperm solution (BD Biosciences, 51–2090KZ). For surface ACE2 staining, cells were chemically detached using accutase or physically scraped off, collected by centrifugation, washed once and incubated in Fc receptor blocking solution at room temperature for 10 min, washed by PBS, and stained with Zombie Aqua dye (BioLegend, 423101) in room temperature, in dark, for 20 min. After washing, cells were incubated on ice in ACE2 antibody (Sino Biological, 10108-RP01) or rabbit IgG (Millipore Sigma, 12-370) at 1:400 ratio for 1 h, followed by incubation in secondary antibody conjugated with Alexa Fluor 488 (Invitrogen, A-11008) at 1:250 ratio, on ice for 1 h , and fixed with BD Cytofix/Cytoperm solution. Cells were then analyzed using Beckman Coulter CytoFLEX S (Beckman Coulter) to the manufacturer’s instructions. Fixed cells were gated based on forward and side scatter for singlets, live cells by live/dead staining, and positive FITC for infected cells. The results were analyzed by FlowJo 10.8.1.

### CFSE cell proliferation assay

MA104 cells were stained following a protocol of CFSE Cell Division Tracker Kit (BioLegend, 423801). The cells proliferated and were harvested at 1–4 days post labeling and analyzed by flow cytometry. Intensity of the FITC signal was shown in histogram and normalized to mode.

#### Confocal microscopy

MA104 cells were seeded in eight-well chamber slides and were fixed when reached 80% confluency in 4% paraformaldehyde for 10 min at room temperature. Cells were then washed with PBS once and stained with WGA (Thermo Scientific, W11262) for 10 min at room temperature. After another wash with PBS, cells were incubated with anti-ACE2 (Sino Biological, 10108-RP01-100) or isotype control (Cell Signaling Technology, 7074S) at room temperature for 1 h. Stained cells were then washed with PBS once and then incubated with the secondary antibody (Invitrogen, A-11008) in dark for another hour. Postsecondary antibody incubation, the cells were washed and stained with DAPI (Invitrogen, P36962). The imaging was performed by a Zeiss LSM880 Confocal Microscope at the Molecular Microbiology imaging core facility at Washington University in St. Louis. Images were analyzed by Velocity v6.3 to generate co-localization and calculate the Pearson correlation coefficients.

#### Cold binding assay

Cells were seeded in 24-well plates and were ready for use when reached 60%–80% confluency. Plates were pre-chilled on ice for 2 ~ 4 hours prior to incubation with VSV-SARS-CoV-2 or SARS-CoV-2. An MOI of 20 was used to ensure maximum viral adsorption. Viruses, mixture of virus with 2B04, a neutralizing antibody against SARS-CoV-2 (16), were incubated at 37°C for 1 h. Pre-incubated virus, mixture of virus, and antibody were chilled on ice for 30 min before added onto pre-chilled cells and incubated on ice. At 1 h post incubation, the cells were washed with pre-chilled PBS three times and then lysed with RLT buffer for RNA harvesting.

#### Human enteroid culture and viral infection

Primary intestinal epithelial cells were derived from ileum biopsy of six patients (211D, 235D, 262D, 217D, 223D, 242D) as previously described (36). Briefly, biopsy was minced with scissors before digestion with dispase. Tissue was strained through a 70-µm filter, and cells were embedded in Matrigel (3D culture) and maintained in 50% L-WRN conditioned medium supplemented with 10 µM each Y-27632 and SB431542 as described previously. For 2D culture, transwell devices with polyester membranes with 0.4 µm pore size were pre-treated with 1:40 Matrigel in PBS for 30 min at 37°C. Cells were trypsinized and filtered before plating on transwell membranes and cultured for 7 days in 50% L-WRN conditioned medium with 10 µM Y-27632. Media were changed in the apical chamber every day and in the basolateral chamber every other day. Differentiation was performed using Dulbecco’s modified Eagle medium/F12 supplemented with 20% FBS, L-glutamine, penicillin/streptomycin, and 10 µM Y-27632 for 3 days before infection. Cells were infected apically with MOI 1 of VSV-SARS-CoV-2 for 1 h at 37°C. Viral supernatant was then replaced with fresh differentiation medium and cells were incubated at 37°C for 24 h before harvest.

#### Statistical analysis

Bar graphs are displayed as means ± SEM. Statistical tests were performed using GraphPad Prism 10.0.2. For ,Fig. 3B through E, H, I, 4A, B, 5, 6C, D and G; Fig. S2D and S4, statistical significance was calculated by Mann Whitney *U* test. For Fig. 6A and B, statistical significance was calculated by two-way ANOVA Šidák’s multiple comparisons test. For Fig. 6E and F; Fig. S5B, statistical significance was calculated by one-way ANOVA Dunnett’s multiple comparisons test. For inhibition and cytotoxicity curves, EC_50_ and CC_50_ values in Fig. 1 and 2; Fig. S1 were calculated using nonlinear regression (curve fit). Asterisks indicate the following: *P ≤ 0.05, **P ≤ 0.01, and ***P ≤ 0.001.

## SUPPLEMENTAL MATERIAL

Fig. S1 mbio.02287-23-s0001.pdf Nitazoxanide effectively inhibits SARS-CoV-2 infection.

Fig. S2 mbio.02287-23-s0002.pdf Clonal CAPN2 KO.

Fig. S3 mbio.02287-23-s0003.pdf Flow cytometry analysis shows reduced VSV-SARS-CoV-2 infection in CAPN2 KO cells.

Fig. S4 mbio.02287-23-s0004.pdf Impact of CAPN2 deletion on Omicron and triple variant infection.

Fig. S5 mbio.02287-23-s0005.pdf Spike cleavage.

Fig. S6 mbio.02287-23-s0006.pdf CAPN2 impacts cell surface ACE2 levels.

Fig. S7 mbio.02287-23-s0007.pdf High CAPN2 expression levels predispose to infection in human ileal enteroids.

Table S1 mbio.02287-23-s0008.pdf SARS-CoV-2 strain mutations

ASM does not own the copyrights to Supplemental Material that may be linked to, or accessed through, an article. The authors have granted ASM a non-exclusive, world-wide license to publish the Supplemental Material files. Please contact the corresponding author directly for reuse.
