# Supplementary material for: Calpain-2 mediates SARS-CoV-2 entry via regulating ACE2 levels
Source: mBio. 2024 Feb 13;15(3):e02287-23. doi: 10.1128/mbio.02287-23 (PMC10936414; doi:10.1128/mbio.02287-23)
Supplement: Fig. S1 — Nitazoxanide effectively inhibits SARS-CoV-2 infection. [file mbio.02287-23-s0001.pdf]

Supplemental figure 1

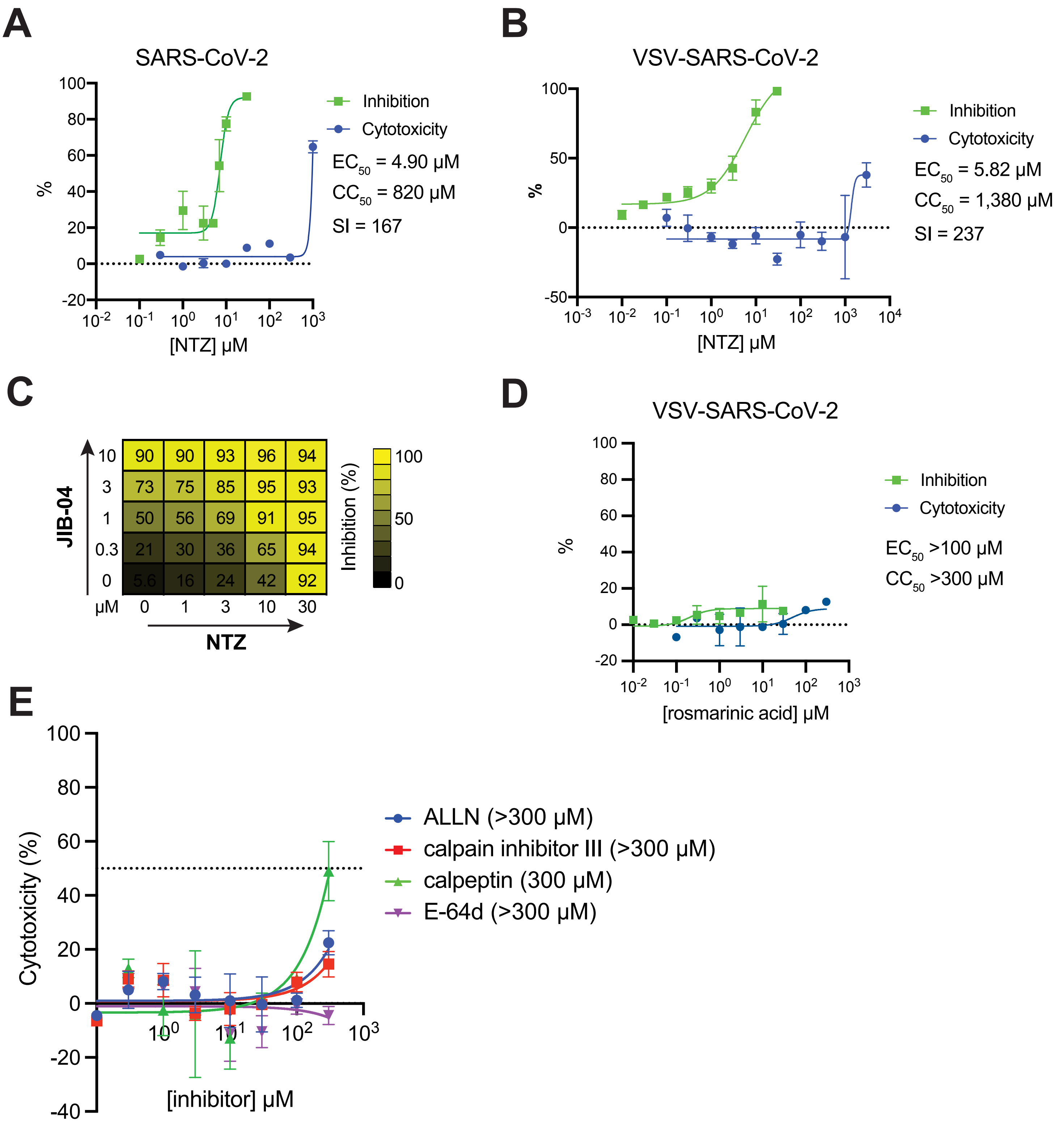

Supplemental Figure 1. Nitazoxanide effectively inhibits SARS-CoV-2 infection

(A) Inhibition and cytotoxicity of nitazoxanide (NTZ) against SARS-CoV-2-mNeonGreen infection. Vero E6 cells were treated with NTZ for 1 h prior to SARS-CoV-2-mNeonGreen infection at an MOI of 0.5. Infection level at 24 hpi was quantified based on immunofluorescence. For cytotoxicity measurement, cells were treated with NTZ at 0.1 μM to 1000 μM for 25 h before being subjected to WST-8 assay to test cell viability. Percentage cell cytotoxicity was plotted as a function of compound dosage. Both assays were repeated three times. EC_{50}, CC_{50} and the SI (selectivity index) are as indicated.

(B) Same as (A) except VSV-SARS-CoV-2 and MA104 cells were used instead.

(C) Drug combination dose-response matrix and VSV-SARS-CoV-2 replication. MA104 cells were treated with NTZ along with JIB-04 at indicated concentrations for 1 h prior to infection by VSV-SARS-CoV-2 at an MOI of 3. GFP signals at 24 hpi were quantified to calculate the percentage of inhibition. Percent inhibition was plotted corresponding to color intensity.

(D) Same as (B) except rosmarinic acid was tested instead.

(E) MA104 cells were treated with ALLN, calpain inhibitor III, calpeptin, and E-64d for 25 hours and tested for cell viability. Percent cytotoxicity was plotted corresponding to dosage. CC_{50} values are as indicated.
