# Supplementary material for: Calpain-2 mediates SARS-CoV-2 entry via regulating ACE2 levels
Source: mBio. 2024 Feb 13;15(3):e02287-23. doi: 10.1128/mbio.02287-23 (PMC10936414; doi:10.1128/mbio.02287-23)
Supplement: Fig. S2 — Clonal CAPN2 KO. [file mbio.02287-23-s0002.pdf]

Supplemental figure 2

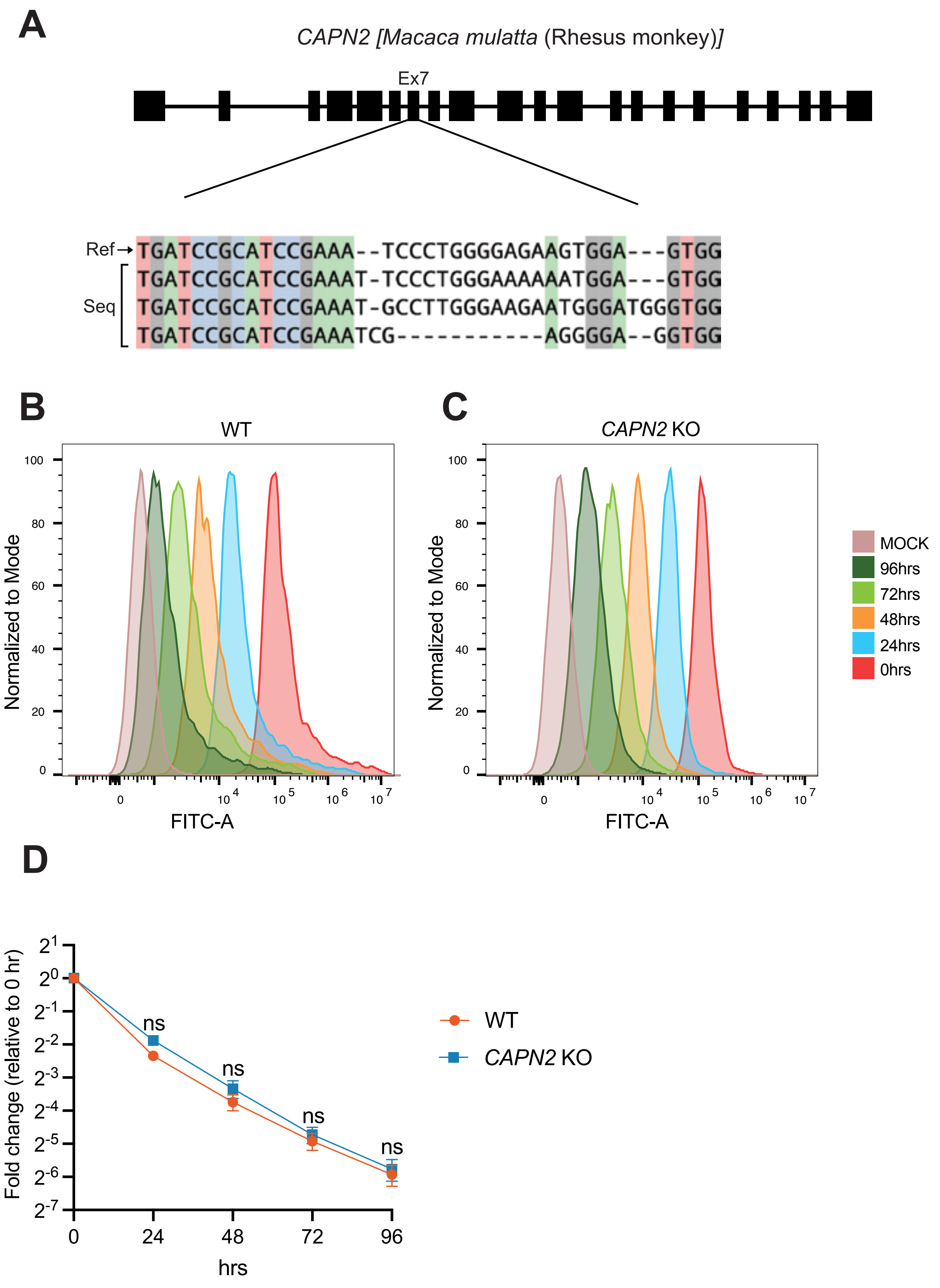

Supplemental Figure 2. Single clonal *CAPN2* KO MA104 cells proliferated comparably to the wild-type cells

(A) sgRNA-targeted exon 7 of the *CAPN2* gene locus in WT (ref) and generated single clone CAPN2 KO (seq) by Sanger sequencing. Conserved and mutated regions were analyzed by CRISPR ID (http://crispid.gbiomed.kuleuven.be/).

(B) CFSE proliferation assay on WT MA104 cells. Cells were stained with CFSE and harvested for flow cytometry at 0, 24, 48, 72 and 96 hours post labeling.

(C) CFSE proliferation assay on *CAPN2* KO MA104 cells. Same protocol was performed as in (B).

(D) CFSE quantified and normalized to fold change based on the signals at 0 hr time point. 4 biological replicates were included.
