# Supplementary material for: Calpain-2 mediates SARS-CoV-2 entry via regulating ACE2 levels
Source: mBio. 2024 Feb 13;15(3):e02287-23. doi: 10.1128/mbio.02287-23 (PMC10936414; doi:10.1128/mbio.02287-23)
Supplement: Fig. S3 — Flow cytometry analysis shows reduced VSV-SARS-CoV-2 infection in CAPN2 KO cells. [file mbio.02287-23-s0003.pdf]

Supplemental figure 3

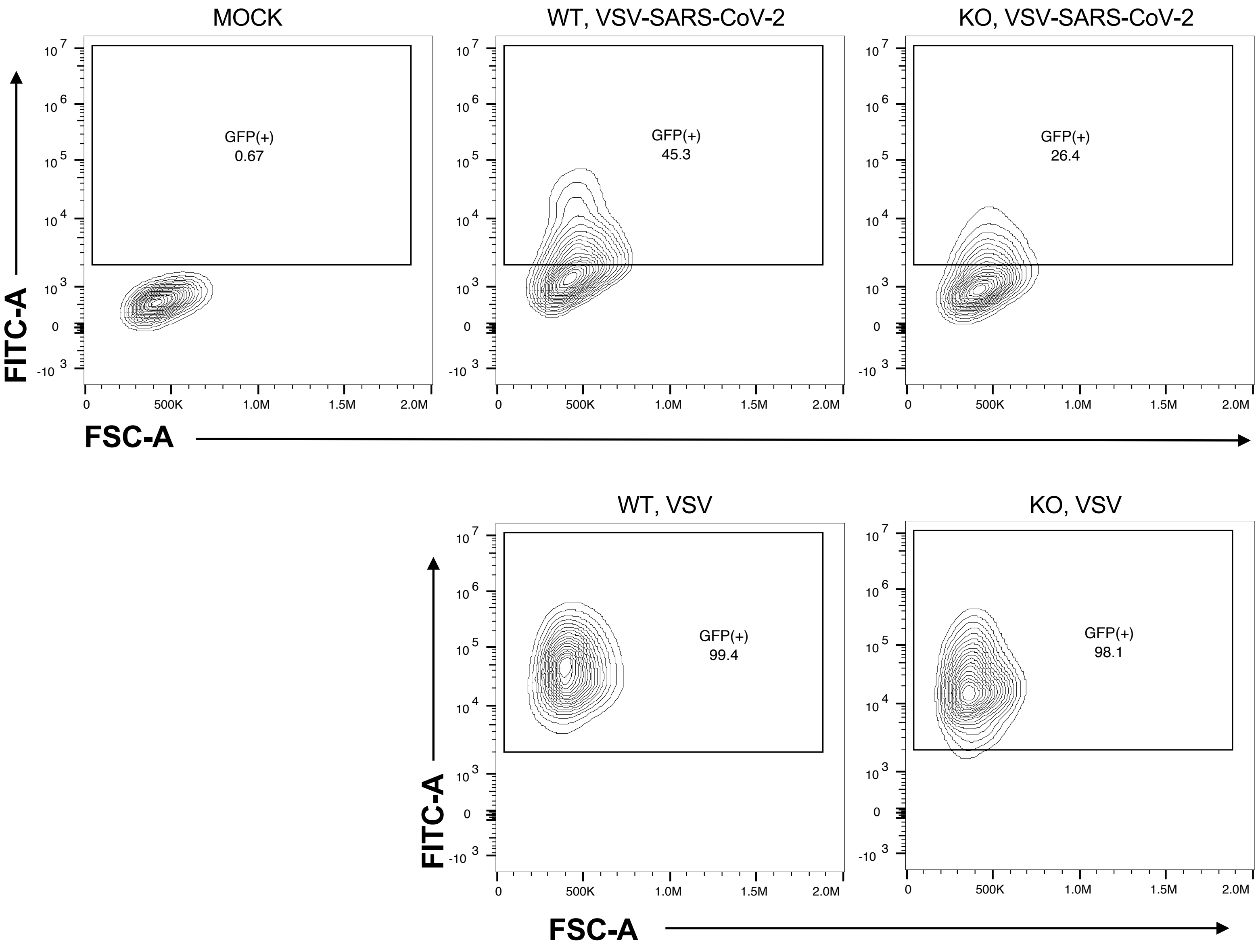

Supplemental Figure 3. VSV-SARS-CoV-2 but not VSV infection was decreased in *CAPN2* KO cells by flow cytometry analysis

Contour plots of WT and KO cells infected with VSV-SARS-CoV-2 and VSV at an MOI of 10 at 8hpi, FITC positive signal was used as an indicator of infection. Percentages of infected cells are shown in the box.
