# Supplementary material for: Calpain-2 mediates SARS-CoV-2 entry via regulating ACE2 levels
Source: mBio. 2024 Feb 13;15(3):e02287-23. doi: 10.1128/mbio.02287-23 (PMC10936414; doi:10.1128/mbio.02287-23)
Supplement: Fig. S4 — Impact of CAPN2 deletion on Omicron and triple variant infection. [file mbio.02287-23-s0004.pdf]

Supplemental figure 4

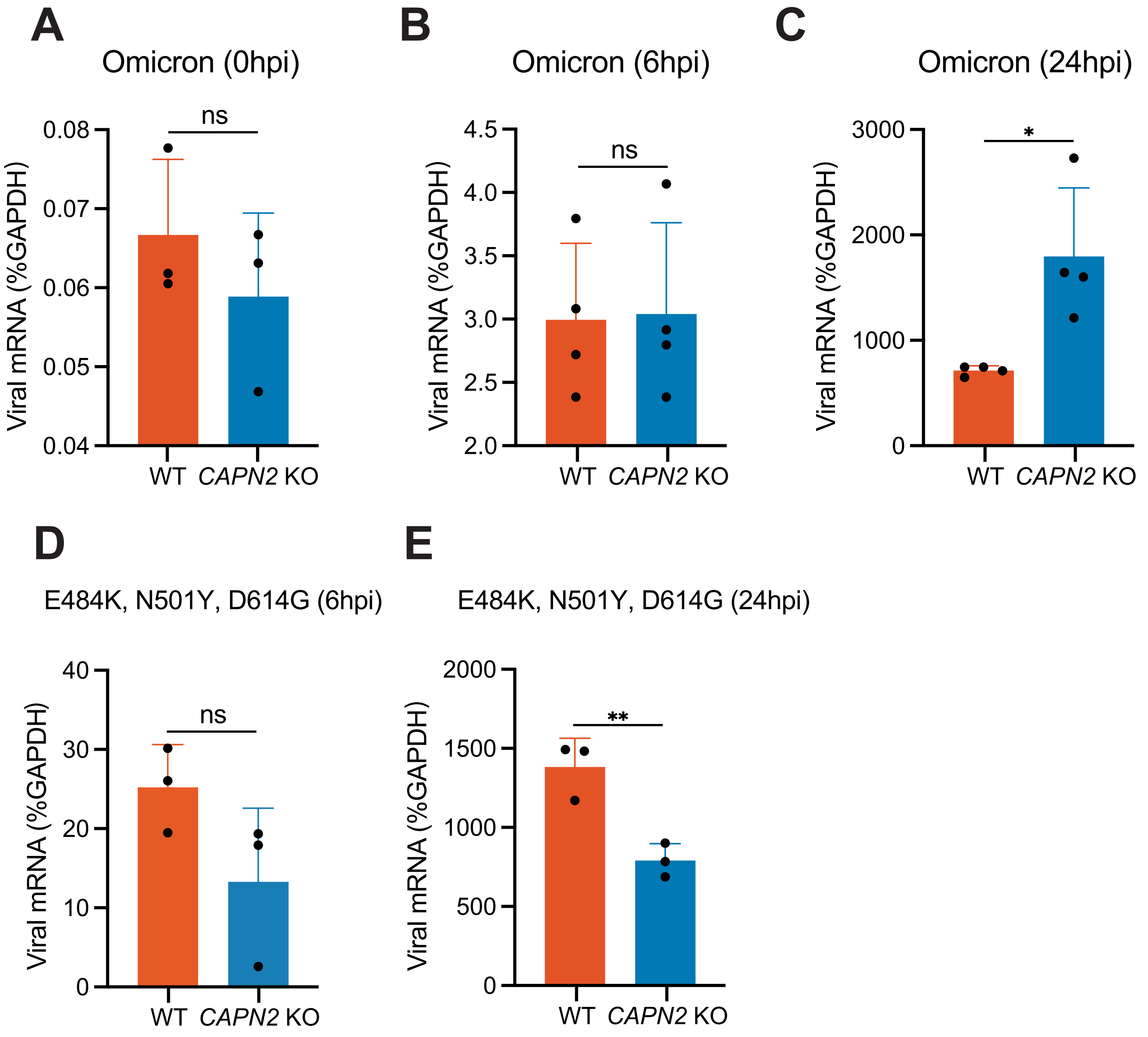

Supplemental Figure 4. CAPN2 deletion minimally impacted infections of SARS-CoV-2 Omicron and a triple spike variant

(A) Viral mRNA production in WT and *CAPN2* KO cells upon infection of Omicron at an MOI of 0.1 and harvested at 0hpi for RNA extraction as the input. Viral mRNA levels were calculated via RT-qPCR and shown as percentage of GAPDH.

(B-C) Same as (A) except harvested at 6hpi and 24hpi, respectively.

(A) Viral mRNA production in WT and *CAPN2* KO cells upon infection of triple spike variant at an MOI of 0.1 and harvested at 6hpi for RNA extraction. Viral mRNA levels were calculated via RT-qPCR and shown as percentage of GAPDH.

(E) Same as (D) except harvested at 24hpi.
