# Supplementary material for: Calpain-2 mediates SARS-CoV-2 entry via regulating ACE2 levels
Source: mBio. 2024 Feb 13;15(3):e02287-23. doi: 10.1128/mbio.02287-23 (PMC10936414; doi:10.1128/mbio.02287-23)
Supplement: Fig. S5 — Spike cleavage. [file mbio.02287-23-s0005.pdf]

Supplemental figure 5

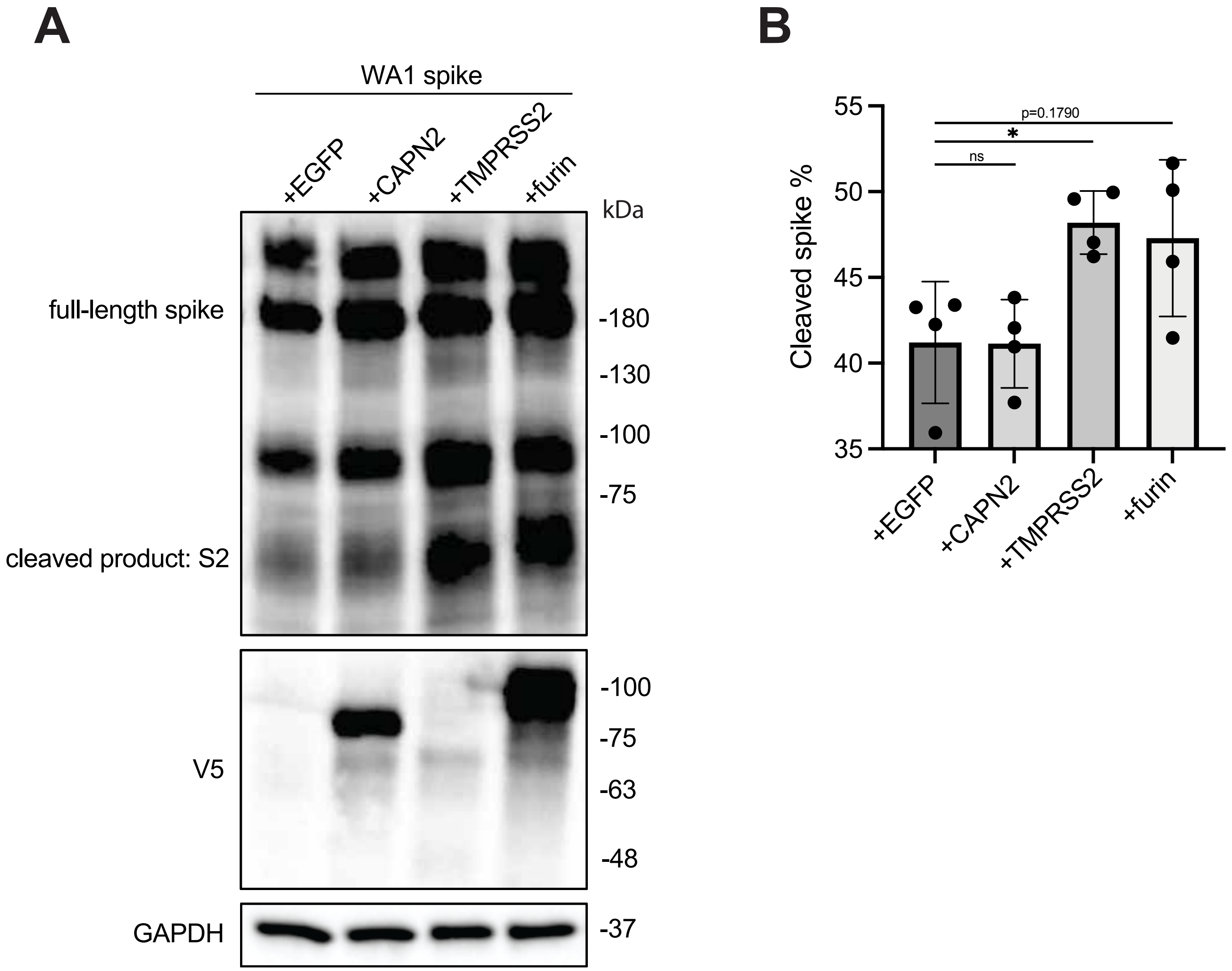

Supplemental Figure 5. WA1 spike is not cleaved significantly by CAPN2

(A) WA1 spike cleavage by CAPN2, TMPRSS2, and furin shown by western blotting. HEK293 cells were co-transfected with 0.5 µg of WA1 spike with 0.5 µg of EGFP, CAPN2, TMPRSS2, and furin, respectively. Protein samples were harvested at 24 hours post transfection. The result is representative of four repeats.

(B) WA1 spike cleavage quantification from 4 repeated experiments described above. Intensities of bands of full-length spike and cleaved product S2 were quantified using ImageJ.
