# Supplementary material for: Calpain-2 mediates SARS-CoV-2 entry via regulating ACE2 levels
Source: mBio. 2024 Feb 13;15(3):e02287-23. doi: 10.1128/mbio.02287-23 (PMC10936414; doi:10.1128/mbio.02287-23)
Supplement: Fig. S6 — CAPN2 impacts cell surface ACE2 levels. [file mbio.02287-23-s0006.pdf]

Supplemental figure 6

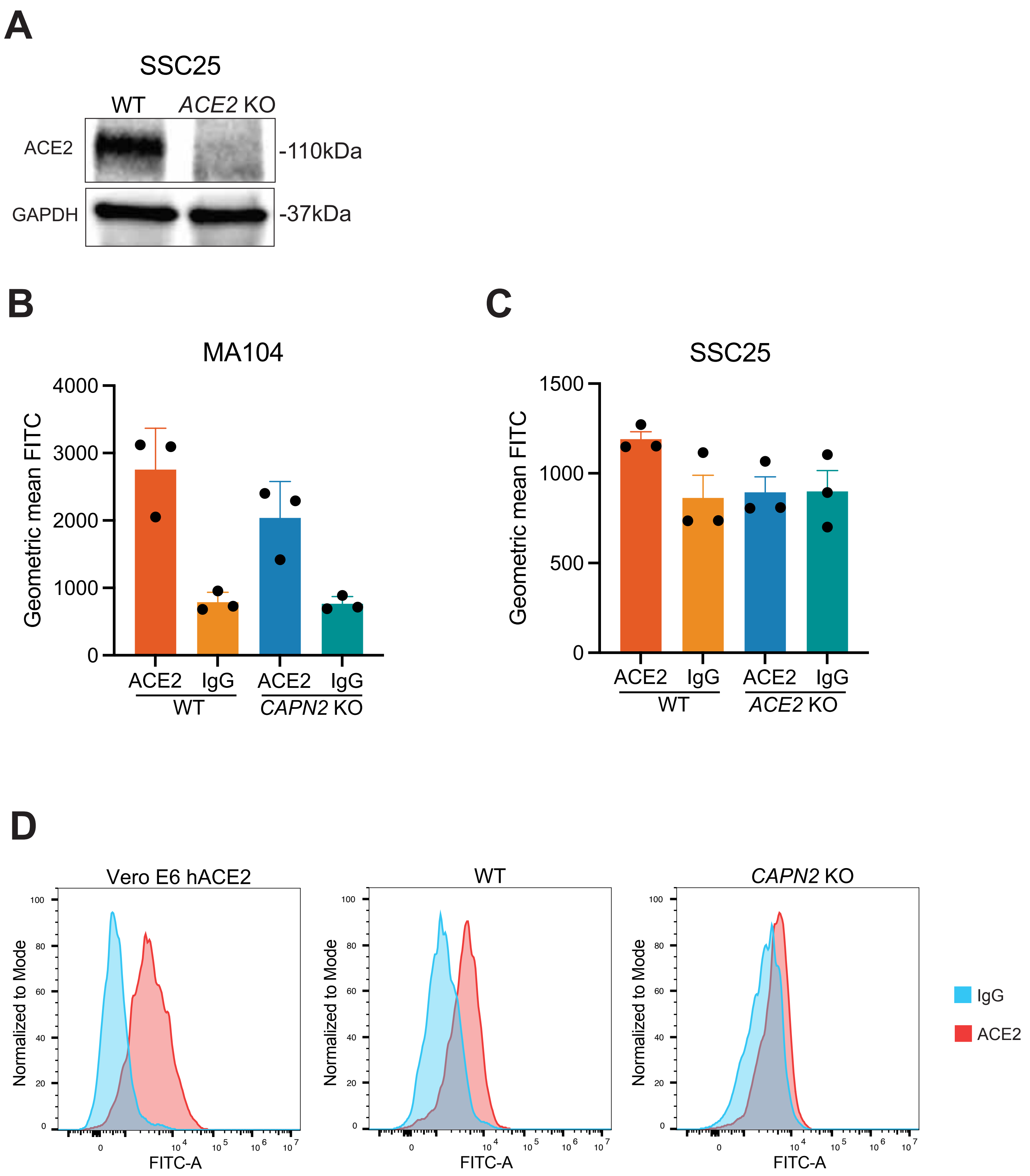

Supplemental Figure 6. Surface ACE2 levels are reduced in *CAPN2* KO cells

(A) Bulk ACE2 protein levels in WT and *ACE2* KO SSC-25 cells. WT and *ACE2* KO SSC-25 cells were harvested for western blot examining the levels of ACE2 and GAPDH.

(B) Mean florescence intensity of surface ACE2 levels of WT and *CAPN2* KO MA104 cells measured and quantified by flow cytometry analysis. Cells were stained with an ACE2 antibody or an IgG rabbit antibody control. Each bar represents 3 biological replicates.

(C) Same as (B) except WT and *ACE2* KO SSC-25 cells were used instead.

(D) Surface ACE2 levels shown by flow cytometry analysis. WT, *CAPN2* KO cells and Vero E6 hACE2 cells were scraped off and stained with an ACE2 antibody or an IgG rabbit antibody control. The FITC signals are shown in histograms.
