# Supplementary material for: Calpain-2 mediates SARS-CoV-2 entry via regulating ACE2 levels
Source: mBio. 2024 Feb 13;15(3):e02287-23. doi: 10.1128/mbio.02287-23 (PMC10936414; doi:10.1128/mbio.02287-23)
Supplement: Fig. S7 — High CAPN2 expression levels predispose to infection in human ileal enteroids. [file mbio.02287-23-s0007.pdf]

Supplemental figure 7

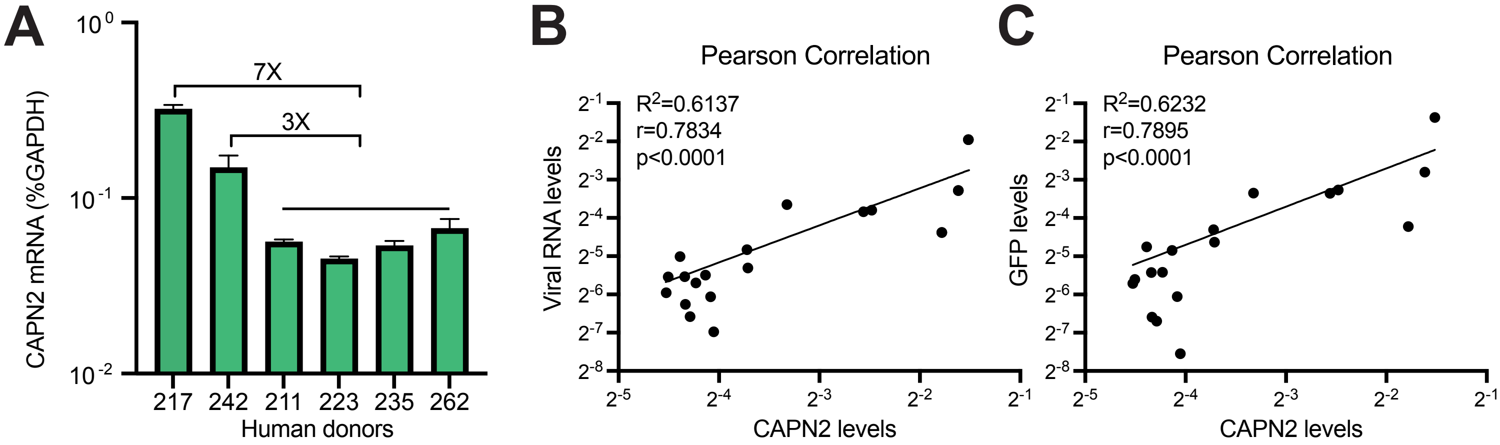

Supplemental Figure 7. High CAPN2 expression levels predispose to infection in human ileal enteroids

(A) CAPN2 expression varies across human enteroid samples. Ileum derived enteroids from six human donors were grown in 2D transwells and apically challenged with VSV-SARS-CoV-2 with an MOI of 1 for 2 hours. RNA isolated after 24 hours was analyzed for CAPN2, VSV-SARS-CoV-2, and GFP by qRT-PCR. Data are the arithmetic mean ± standard error of the mean from one experiment performed in triplicate.

(B) CAPN2 expression positively correlates with viral VSV-N levels. Statistically significant correlations were determined by Pearson correlation coefficient test and a trend line was calculated by linear regression and presented in the graph ($P < 0.0001$).

(C) CAPN2 expression positively correlates with the virus GFP reporter. Statistically significant correlations were determined by Pearson correlation coefficient test and a trend line was calculated by linear regression and presented in the graph ($P < 0.0001$).
