# Supplementary material for: Calpain-2 mediates SARS-CoV-2 entry via regulating ACE2 levels
Source: mBio. 2024 Feb 13;15(3):e02287-23. doi: 10.1128/mbio.02287-23 (PMC10936414; doi:10.1128/mbio.02287-23)
Supplement: Table S1 — SARS-CoV-2 strain mutations [file mbio.02287-23-s0008.pdf]

Supplemental table 1

WA1	Delta B.1.617.2	Omicron B.1.1.529
No spike mutations	T19R, G142D, E156G, F157del, R158del, L452R, T478K, D614G, P681R, F855S, D950N, C1235F	T95I, G142D, V143del, Y144del, Y145del, N211I, L212del, G339D, S371L, S373P, S375F, K417N, N440K, G446S, S477N, T478K, E484A, Q493R, G496S, Q498R, N501Y, Y505H, T547K, D614G, H655Y, N679K, P681H, N764K, D796Y, N856K, Q954H, N969K, L981F, D1146

Supplemental Table 1. Spike Mutations in SARS-CoV-2 variants
